# Association between menopause-related symptoms and muscle mass index among perimenopausal and postmenopausal women and the mediating role of estrogen levels

**DOI:** 10.3389/fendo.2025.1628612

**Published:** 2025-07-23

**Authors:** Xiaoyi Wang, Dongjian Yang, Jiahui Li, Lei Jin, Shuhua Xia, Furui Jin

**Affiliations:** ^1^ International Peace Maternity and Child Health Hospital, School of Medicine, Shanghai Jiao Tong University, Shanghai, China; ^2^ Shanghai Key Laboratory of Embryo Original Diseases, Shanghai, China; ^3^ Department of Gynecology, Shanghai Fengcheng Hospital, Shanghai, China

**Keywords:** peri- and post-menopause, menopause-related symptoms, muscle mass index, estradiol, mediation

## Abstract

**Background:**

The decline in muscle mass is a common concern among perimenopausal women. However, the association between menopause-related symptoms and muscle mass remains inconclusive, and the mechanistic role of estrogen is still unclear.

**Methods:**

The study included 407 peri- and postmenopausal women aged 40–60 years who visited the International Peace Maternity and Child Health Hospital. Menopausal symptoms were assessed using the modified Kupperman Index (KMI). Muscle mass was evaluated using the InBody 270 analyzer, and sex hormone levels were determined by chemiluminescent immunoassay. Multiple linear regression and Mediation analysis were conducted to examine the association of KMI with MMI and the mediation of estrogen.

**Results:**

A total of 407 valid cases were collected. The mean age of the patients was 49.96 ± 3.25 years, with an average body weight of 58.02 ± 7.36 kg and an average BMI of 22.50 ± 2.61 kg/m². The findings showed that advanced age, lower education level, and reduced muscle mass index (MMI) were linked to elevated KMI scores (p<0.05). Patients with hypertension had higher KMI scores (p<0.05). Additionally, decreased estradiol (E_2_) levels correlated with heightened menopausal symptoms (p<0.05). After controlling for confounding factors such as age, educational level, menopausal stage, history of hypertension, follicle-stimulating hormone (FSH), and E_2_, KMI was negatively correlated with MMI (β=-1.612, 95% CI: -2.677 to -0.546, p=0.003). Specifically, for each unit increase in MMI, KMI decreased by 1.612 points (R²=0.186, p=0.003). Stratified analysis showed that the negative correlation between KMI and MMI was significant only in premenopausal women. Both the direct and indirect effects of MMI and E_2_ on KMI were statistically significant (p<0.01). The mediating effect of MMI on KMI through E_2_ accounted for 26.9% (p=0.001).

**Conclusions:**

Lower muscle mass is associated with severe menopausal symptoms, partially mediated by estrogen. Maintaining muscle mass may alleviate symptoms, highlighting the importance of resistance training and hormone regulation in perimenopausal women. However, due to the cross-sectional nature of the study, causality cannot be inferred. Longitudinal or interventional studies are warranted to further validate these associations and explore underlying mechanisms.

## Introduction

With the increasing life expectancy of women worldwide, the peri- and postmenopausal female population is rapidly growing. It is estimated that by 2050, the number of women aged 50 and above will increase from 1 billion in 2020 to 1.6 billion (1). Menopause, typically occurring around age 51, affects most women, with symptoms lasting an average of seven years (2). Over 35 menopause-related symptoms have been identified, including hot flashes, sweating, sleep disturbances, anxiety, mood swings, and decreased libido (3, 4), but musculoskeletal symptoms are often overlooked. Studies show about 70% of middle-aged women experience musculoskeletal syndromes during peri- and postmenopause (5, 6), leading to muscle mass decline, impaired function, increased fall risk, and reduced quality of life.

The muscle mass of postmenopausal women decreases by approximately 0.6% per year (7), and if not addressed early, this can lead to severe long-term health consequences. Early detection and management of muscle loss and menopausal symptoms are therefore crucial in clinical practice.

Existing studies suggest that menopause-related symptoms may be associated with lean body mass (8). International research has also demonstrated that reduced muscle mass correlates with more severe vasomotor symptoms (9, 10). However, an Indian study reported no significant correlation between quality of life and sarcopenia in postmenopausal women (11), indicating that these associations may vary across populations. Estradiol (E_2_), the most biologically active estrogen, affects bones, tendons, muscles, cartilage, ligaments, and adipose tissue (12, 13), suggesting a potential role in the link between menopausal symptoms and muscle mass. However, research on this relationship remains limited, and the specific mechanisms are still unclear.

Therefore, this study investigates the association between menopause-related symptoms and muscle mass in Chinese peri- and postmenopausal women, focusing on the mediating role of hormone levels. The findings aim to provide evidence for the early management of muscle loss and improve health care strategies for perimenopausal women.

## Methods

### Study population

**Figure 1:**
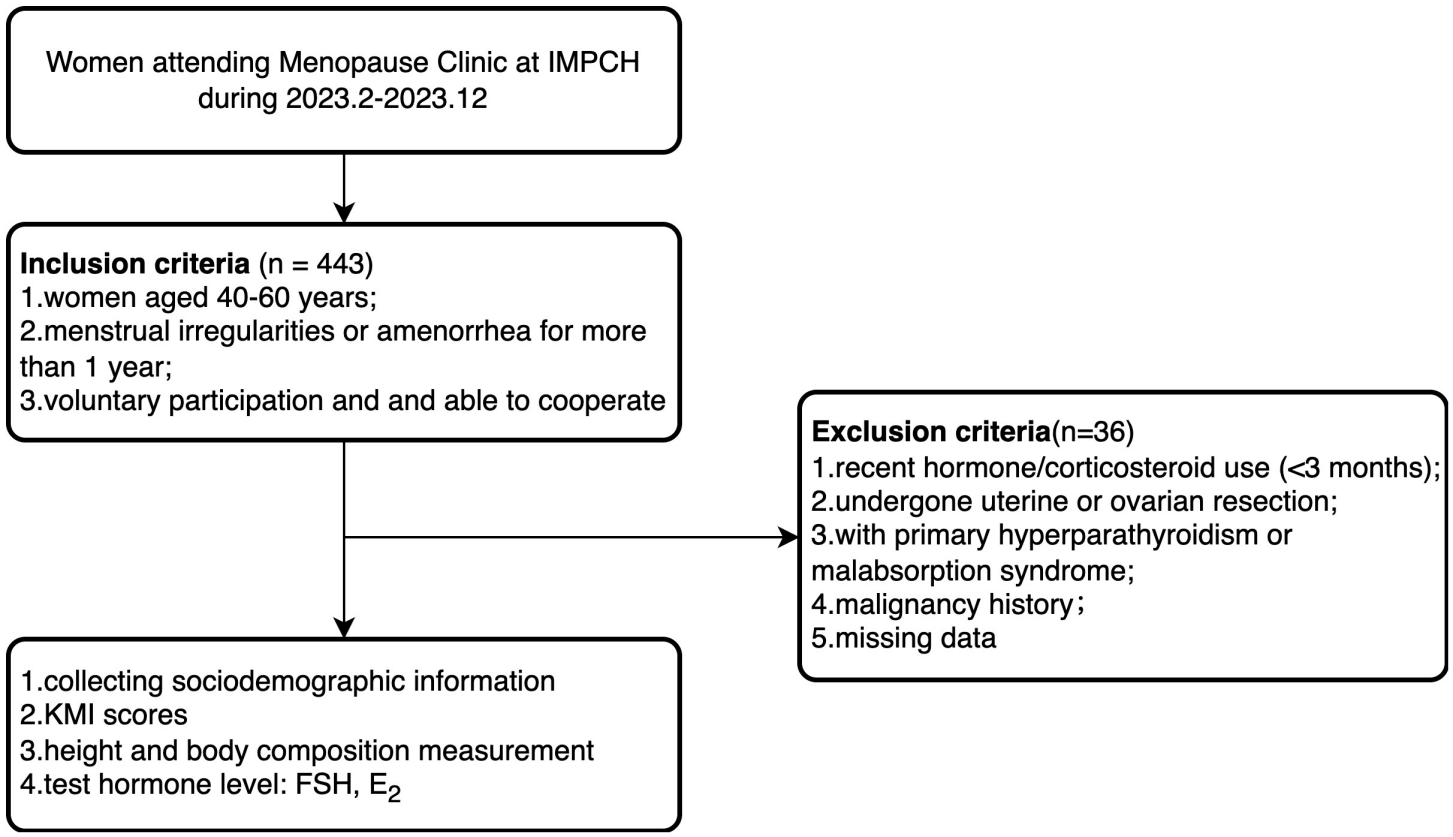
Study flowchart.

The study selected peri- and postmenopausal women who visited the Menopause Clinic at the International Peace Maternity and Child Health Hospital (IPMCH) from February 2023 to December 2023. The inclusion criteria were: (1) age between 40–60 years; (2) menstrual irregularities (variation in cycle length ≥7 days in the past 10 menstrual cycles) or amenorrhea for more than 1 year; (3) voluntary participation and ability to actively cooperate with the survey. The exclusion criteria were: (1) patients who had received hormone replacement therapy or corticosteroid treatment in the past 3 months; (2) patients who have undergone uterine or ovarian resection; (3) patients with primary hyperparathyroidism or malabsorption syndrome; (4) patients with a history of malignant tumors; (5) missing data (**Figure 1**). This study included 407 eligible participants and received approval from the Medical Ethics Committee of the IPMCH (No.: GKLW-2023-001), and all participants signed informed consent forms.

### Covariates

Demographic and socioeconomic information was collected through medical history inquiries, including age, menstrual status, education level, occupation type, income level, marital status, number of births, number of miscarriages, and past medical history, all of which were recorded in each patient’s medical file. Using the Stages of Reproductive Aging Workshop +10 (STRAW +10) criteria (14), participants were classified into four groups: early menopausal transition (variation in cycle length ≥7 days, occurring repeatedly within 10 menstrual cycles), late menopausal transition (cycle length ≥60 days), menopause for 1–2 years, and menopause for more than 2 years. Education levels were categorized into three groups: middle school or below, university, and master’s degree or above. Occupation types were classified into three categories: manual labor, light physical labor, and intellectual work. Income levels were divided into four groups: below 5,000 CNY per month, 5,000–10,000 CNY, 10,000–20,000 CNY, and above 20,000 CNY.

### Outcomes

Menopausal symptoms were evaluated using the modified Kupperman Index (15), which was widely used in clinical and research settings in China to evaluate menopausal symptoms (16–18). Menopausal Symptom Severity Classification: normal: KMI score ≤6; mild: 6 < KMI score ≤15; moderate: 16 < KMI score ≤30; severe: KMI score >30. The KMI consists of two components for each symptom: a base score and a severity score. Base Score: hot flashes and sweating: 4 points; paresthesia, insomnia, depression and suspicion, dyspareunia, mood swings, and urinary system symptoms: 2 points each; dizziness, osteoarticular pain, fatigue, palpitations, headache, and formication: 1 point each. Severity Score Criteria: no symptoms: 0 points; occasional symptoms: 1 point; frequent symptoms: 2 points; frequent and severe symptoms: 3 points. KMI score = Basic score × Severity score.

### Measurement of muscle mass and hormones

Body composition was measured using the InBody 270 analyzer, including weight, waist-to-hip ratio, and muscle mass. Each measurement covered the upper limbs, lower limbs, and trunk. Before measurement, participants were required to remove shoes and socks, wear lightweight clothing, and empty their pockets of any belongings. They stood barefoot on the electrodes, held the handles with their thumbs placed on the upper part and the other four fingers gripping below, with arms extended straight. The staff recorded each participant’s name, date of birth, and height before obtaining the measurement results. Calculation of Muscle Mass Index (MMI): MMI = Muscle Mass/Height² (19).

Sex hormone levels, including FSH and E_2_, were measured using the chemiluminescent immunoassay method (Beckman Coulter DXI800, USA). For postmenopausal women, hormone levels were measured at random. For premenopausal women, hormone levels were measured on days 2–5 of the menstrual cycle or, if menstruation had been absent for more than 40 days, immediately after ruling out pregnancy.

### Statistical methods

Continuous variables were expressed as mean ± standard deviation (SD), while categorical variables were presented as frequencies and percentages. Independent sample t-tests were used to compare normally distributed variables with homogeneity of variance. The Mann-Whitney U test was applied for variables that did not meet normality or homogeneity of variance assumptions. Categorical variables were compared using the chi-square test. Multiple linear regression was performed to analyze the relationship between KMI and MMI. Mediation analysis was conducted using AMOS 23.0, with estrogen levels log-transformed for analysis. The mediation effect was tested using the Bootstrap method. Data were analyzed using SPSS 24.0 (SPSS, Inc., Chicago, IL, USA), and a two-tailed P-value <0.05 was considered statistically significant.

## Results

### Comparison of baseline characteristics among patients with different menopausal symptom severity

A total of 407 valid cases were included in this study. The average age of the patients was 49.96 ± 3.25 years, with an average weight of 58.02 ± 7.36 kg and an average BMI of 22.50 ± 2.61 kg/m². Based on the severity of menopausal symptoms, participants were categorized into two groups: KMI ≤15 (n = 194) and KMI > 15 (n = 213). Patients with higher age, lower education levels, and lower MMI had significantly elevated KMI scores (p<0.05). Patients with hypertension had elevated KMI scores (p<0.05). Higher FSH levels and lower E_2_ levels were associated with more intense menopausal symptoms (p<0.05). Details are presented in **Table 1**.

**Table 1:** Comparison of baseline characteristics among patients with different menopausal symptom severity.

Variable	All cases (n = 407)	KMI (points)		P Value
		≤15(n=194)	>15(n=213)	
Age (years)	49.96 ± 3.25	49.26 ± 3.28	50.59 ± 3.10	<0.001
Height (m)	160.54 ± 4.69	160.55 ± 4.99	160.52 ± 4.40	0.947
Weight (kg)	58.02 ± 7.36	58.42 ± 7.45	57.66 ± 7.28	0.301
BMI (kg/m^2^)	22.50 ± 2.61	22.65 ± 2.66	22.36 ± 2.57	0.264
Waist-to-hip ratio	0.87 ± 0.05	0.87 ± 0.05	0.87 ± 0.04	0.070
Body fat percentage (%)	30.90 ± 5.40	30.60 ± 5.35	31.17 ± 5.45	0.287
Muscle Mass Index (kg/m²)	8.35 ± 0.74	8.47 ± 0.75	8.25 ± 0.71	0.002
Menopausal Status, n (%)				<0.001
Early menopausal transition	116(28.50)	65(33.51)	51(23.94)	
Late menopausal transition	171(42.01)	91(46.91)	80(37.56)	
Menopause 1–2 years	87(21.38)	27(13.92)	60(28.17)	
Menopause >2 years	33(8.11)	11(2.67)	22(10.33)	
Education Level, n (%)				0.009
Middle school or below	109(26.8)	42(21.65)	67(31.46)	
University	245(60.2)	118(60.82)	127(59.62)	
Master’s degree or above	53(13.0)	34(17.53)	19(8.92)	
Occupation Type, n (%)				0.573
Mental labor	243(59.71)	121(62.37)	122(57.28)	
Light physical labor	111(27.27)	49(25.26)	62(29.11)	
Physical labor	53(13.02)	24(12.37)	29(13.62)	
Marital Status, n (%)				0.722
Married	367(90.17)	176(90.72)	191(89.67)	
Single/Divorced/Widowed	40(4.83)	18(9.28)	22(10.33)	
Income (CNY/month), n (%)				0.277
<5000	26(6.39)	12(6.19)	14(6.57)	
5000-10000	148(36.36)	68(35.05)	80(37.56)	
10000-20000	135(33.17)	59(30.41)	76(35.68)	
>20000	98(24.08)	55(28.35)	43(20.19)	
Number of Births, n (%)				0.559
0	28(6.9)	11(5.67)	17(7.98)	
1	337(82.8)	161(82.99)	176(82.63)	
≥2	42(10.3)	22(11.34)	20(9.39)	
Hypertension, n (%)				0.037
Yes	33(8.1)	10(5.15)	23(10.8)	
No	374(91.2)	184(94.85)	190(89.2)	
Diabetes, n (%)				0.869
Yes	12(2.95)	6(3.09)	6(2.82)	
No	395(97.05)	188(96.91)	207(97.18)	
FSH (IU/L)	61.00(28.60,83.50)	50.65(20.90,76.28)	68.20(45.50,89.20)	<0.001
E_2_ (pmol/L)	49.00(27.00,168.00)	80.50(33.75,261.25)	37.00(25.00,84.00)	<0.001

### Multiple linear regression analysis

In the unadjusted Model 1, KMI was significantly negatively correlated with MMI (β = -2.088, 95% CI: -3.156 to -1.020, p < 0.001), with an adjusted R² of 0.035. In Model 2, after adjusting for age, education level, menopausal status, and history of hypertension, KMI remained negatively correlated with MMI (β=-2.083, 95% CI: -3.131 to -1.035, p=0.000), and the adjusted R² increased to 0.150. In Model 3, after further adjusting for FSH, and E_2_, the negative correlation between KMI and MMI persisted (β = -1.612, 95% CI: -2.677 to -0.546, p=0.003), indicating that for each unit increase in MMI, KMI decreased by 1.612. The adjusted R² increased to 0.186, suggesting that hormone levels may play a mediating role in the relationship between KMI and MMI (**Table 2**). However, when we conducted a stratified analysis of perimenopausal women based on menopausal status, we found a significant negative correlation between KMI and MMI in premenopausal women, whereas this association was not significant in postmenopausal women. For premenopausal women, after adjusting for age, education level, menopausal status, and history of hypertension, KMI remained negatively correlated with MMI (β=-2.193, 95% CI: -3.400 to -0.986, p=0.000), and the adjusted R² increased to 0.072. After further adjusting for FSH, and E2, the negative correlation between KMI and MMI persisted (er -1.694, 95% CI: -2.924 to -0.464, p=0.008) (**Table 3**).

**Table 2:** Regression models for the relationship between muscle mass index and menopausal symptoms.

Regression model	KMI		
	Adjusted R^2^	β Value (95% CI)	P Value
Unadjusted model	0.035	-2.088(-3.156∼-1.020)	0.000
Adjusted Model 1 ^a^	0.150	-2.083(-3.131∼-1.035)	0.000
Adjusted Model 2 ^b^	0.186	-1.612(-2.677∼-0.546)	0.003

a : Adjusted for age, education level, hypertension history, and menopausal stage.

b : Adjusted for FSH and E_2_ in addition to the adjustments in Adjusted Model 1.

**Table 3:** Regression models of the relationship between muscle mass index and menopausal symptoms stratified by menopausal stage.

Regression model	KMI		
	Adjusted R^2^	β Value (95% CI)	P Value
Premenopausal women			
Unadjusted model	0.025	-1.716(-2.891∼-0.541)	0.004
Adjusted Model 1 ^a^	0.072	-2.193(-3.400∼-0.986)	0.000
Adjusted Model 2 ^b^	0.115	-1.694(-2.924∼-0.464)	0.007
Menopausal women			
Unadjusted model	0.017	-1.967(-4.204∼-0.269)	0.084
Adjusted Model 1 ^a^	0.050	-1.943(-4.172∼-0.286)	0.087
Adjusted Model 2 ^b^	0.059	-1.567(-3.827∼-0.694)	0.172

a : Adjusted for age, education level, and hypertension history.

b : Adjusted for FSH and E_2_ in addition to the adjustments in Adjusted Model 1.

### Mediation effect analysis

**Figure 2:**
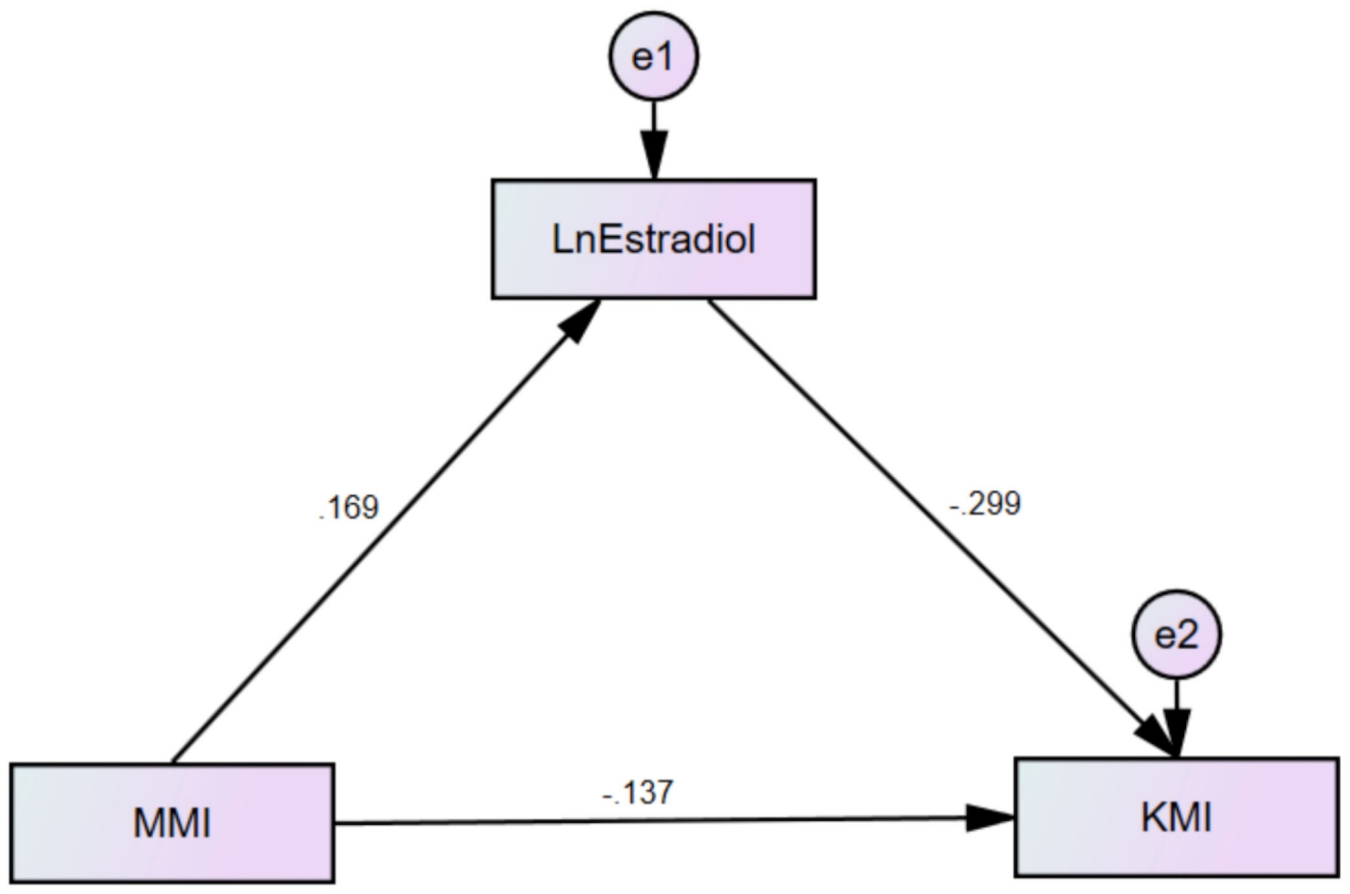
Mediation of estradiol on association of MMI with KMI.

In this study, KMI was defined as the dependent variable, MMI as the independent variable, and E_2_ as the mediating variable. Based on this, a mediation effect model was constructed (**Figure 2**). The significance of the mediation effect was tested using the Bootstrap method. The findings indicated that MMI and E_2_ had both significant direct and indirect effects on KMI (p<0.01). Specifically, E_2_ mediated 26.9% of MMI’s impact on KMI, as presented in **Table 4**.

**Table 4:** Mediation of estradiol on association of MMI with KMI.

Effect	Path	β Value	P Value	95%CI	
				Lower	Upper
Indirect Effect	MMI→E_2_→KMI	-0.562	0.001	-0.993	-0.223
Direct Effect	MMI→KMI	-2.088	0.000	-3.061	-1.110
R	–	0.269	0.002	0.104	0.549

## Discussion

Our study explored the relationship between menopausal symptoms and MMI and analyzed the mediating role of E_2_ levels. The results showed a significant negative correlation between KMI and MMI, with E_2_ playing a partial mediating role between the two. This finding provides new evidence for understanding the relationship between muscle mass decline and menopausal symptoms in women during the perimenopausal and postmenopausal stages.

Menopausal symptoms are complex and influenced by numerous factors. This study is the first to identify a significant negative correlation between KMI and MMI, with MMI being an independent protective factor for moderate to severe menopausal symptoms, regardless of age, education level, menopausal stage, hypertension history, and levels of FSH and E_2_. At present, studies on the effects of body composition on menopausal symptoms are scarce. Previous studies have suggested that lean body mass, particularly in the trunk, serves as a protective factor for moderate to severe menopausal symptoms (8). In addition, a study from Korea showed that lower grip strength (a commonly used indicator for diagnosing sarcopenia) is positively correlated with higher KMI scores (9). When assessing menopausal symptoms such as vasomotor symptoms (VMS) alone, some studies have also found a significant correlation between sarcopenia and VMS (10). However, some studies have reported an inverse correlation between the prevalence of sarcopenia and the incidence of VMS, while the paraspinal muscle index shows a positive correlation with the incidence of VMS (20). Moreover, a higher body fat percentage in perimenopausal women is strongly linked to increased severity of menopausal symptoms (2, 21). Notably, in our stratified analysis, this relationship was more pronounced in premenopausal women, where the negative association between KMI and MMI remained significant even after adjusting for confounding variables and hormone levels, suggesting that muscle mass may play a more prominent role in influencing menopausal symptoms prior to the onset of menopause. Although our study provides new evidence on the link between muscle mass and menopausal symptoms, longitudinal data remain limited. Recent cohort studies from Canada and Switzerland indicate that sarcopenia incidence increases with age in postmenopausal women and is influenced by hormone therapy and physical activity (22, 23). These findings highlight the need for future longitudinal research to clarify causal relationships and guide personalized intervention strategies.

In addition, this study found that older women and those with lower educational levels had higher KMI (P<0.05), which is consistent with previous research (24, 25). This may be due to higher-educated women having better access to perimenopausal health information and managing symptoms more effectively through self-regulation.

Notably, this study found that menopausal symptoms were more severe in patients with hypertension, while no significant association was observed between diabetes and KMI. Previous studies have shown that systolic blood pressure, diastolic blood pressure, and dyslipidemia are positively correlated with menopausal quality of life scores, whereas fasting blood glucose and high-density lipoprotein have no significant relationship with total menopausal symptom scores (26). The decline in estrogen levels may lead to metabolic abnormalities, vasomotor dysfunction, and muscle mass loss, thereby exacerbating menopausal symptoms. Additionally, the occurrence of vasomotor symptoms is associated with an increased incidence of cardiovascular events. Studies have indicated that more frequent vasomotor symptoms are correlated with higher blood pressure, and compared to women without vasomotor symptoms, those experiencing vasomotor symptoms may be more susceptible to developing hypertension (27).

Further analysis of the mediating role of E_2_ between MMI and KMI revealed that patients with higher FSH levels and lower E_2_ levels had lower MMI (P<0.05). Moreover, E_2_ played a significant mediating role in the relationship between MMI and KMI (P<0.01). This finding supports the potential mechanism of estrogen in muscle mass changes during the (peri)menopausal period. Estrogen not only acts as an inflammation regulator but also inhibits the release of the inflammatory cytokine TNF-α (28), TNF-α contributes to muscle protein breakdown and impairs the muscle’s capacity for repair (29), additionally, TNF-α is released by adipocytes, promoting fat accumulation and impairing muscle function (30–32). Declining ovarian function may directly adversely affect muscle mass through estrogen receptors in muscle and joint tissues (33). Satellite cells are stem cells located on muscle fibers, responsible for promoting muscle plasticity and regeneration (34). Satellite cells are stem cells located on muscle fibers, responsible for promoting muscle plasticity and regeneration (35).

This study has several strengths, including comprehensive data collection and the inclusion of key hormonal and body composition indicators. The statistical models were rigorously adjusted for major demographic and clinical variables. However, several limitations should be noted. First and foremost, the cross-sectional design of this study inherently limits the ability to establish causal relationships. Consequently, the temporal sequence among menopausal symptoms, hormone levels, and muscle mass cannot be determined, which restricts causal inference. Although confounding variables were considered, residual confounding may persist, particularly from unmeasured factors such as physical activity, dietary intake, sleep quality, and comorbidities, all of which are well-known to influence both muscle mass and menopausal symptoms. The absence of data on these key lifestyle factors represents an important limitation of this study and should be addressed in future research. In addition, while blood samples were collected at standardized times to minimize hormonal variability, a single estradiol measurement may not reflect true baseline levels due to diurnal fluctuations and cycle irregularity, especially in perimenopausal women. This could attenuate observed associations between E2 and muscle mass. Finally, although bioelectrical impedance analysis using the InBody 270 is practical and widely used in field settings, it may overestimate skeletal muscle mass due to its sensitivity to hydration and reliance on predictive equations (36, 37). Future longitudinal or interventional studies with repeated assessments and broader population samples are warranted to validate these findings and explore the underlying mechanisms in more depth. Despite these limitations, our study adds to the growing body of evidence supporting the link between hormonal changes, muscle mass, and menopausal symptoms, and underscores the importance of early monitoring and intervention in perimenopausal women.

## Conclusion

This study found a negative correlation between KMI and MMI, with E2 partially mediating this relationship. These findings highlight the potential importance of considering muscle mass changes in perimenopausal health assessments. Early identification of reduced muscle mass can not only inform future intervention strategies such as exercise and hormonal therapy but also emphasizes the need for methods of early detection, such as regular body composition analysis and grip strength testing. Additionally, possible therapeutic measures including tailored exercise programs, nutritional supplementation, and hormone therapy warrant further exploration and application. These data could help promote awareness campaigns that, in the long term, may improve the quality of life for postmenopausal women. Nevertheless, the clinical application of personalized interventions should be interpreted cautiously and requires validation through longitudinal or interventional studies.

## Data Availability

The raw data supporting the conclusions of this article will be made available by the authors, without undue reservation.
